# Impact of Covid-19 pandemic on tuberculosis mortality in Brazil: a time series analysis

**DOI:** 10.11606/s1518-8787.2026060007226

**Published:** 2026-03-16

**Authors:** Bernardo Bastos Wittlin, Felipe Bezerra Pimentel Araújo, Antônio Augusto Moura da Silva

**Affiliations:** I Universidade Federal do Maranhão. Programa de Pós-Graduação em Saúde Coletiva. São Luís, MA, Brasil; II Universidade Federal do Maranhão. Programa de Pós-Graduação em Engenharia Elétrica. São Luís, MA, Brasil; III Universidade Federal do Maranhão. Departamento de Saúde Pública. São Luís, MA, Brasil

**Keywords:** Tuberculosis, Mortality, Interrupted Time Series Analysis, COVID-19

## Abstract

**OBJECTIVE:**

To evaluate changes in the trend of tuberculosis mortality in Brazil over recent decades and assess the impact of the Covid-19 pandemic on this indicator.

**METHODS:**

We analyzed the national and regional time series of tuberculosis mortality from 2000 to 2023 using joinpoint regression. To assess the pandemic’s impact, we applied two interrupted time series (ITS) approaches: segmented linear regression and the AutoRegressive Integrated Moving Average with eXogenous variables (ARIMAX model). We also used Autoregressive Integrated Moving Average (ARIMA) modeling to estimate excess tuberculosis deaths linked to the pandemic and to forecast mortality trends through 2030.

**RESULTS:**

An increase in tuberculosis mortality was observed starting in 2021, reaching 2.4 deaths per 100 thousand people in both 2022 and 2023 — similar to rates observed in 2011. This represents a reversal in the declining trend seen throughout the 2000s and 2010s, affecting all macroregions. The annual percentage change from 2019 to 2023 was +6.5% (95% confidence interval — 95%CI 4.42 to 9.98), contrasting with an average decline of -1.93% (95%CI -2.19 to -1.69) over the full period. Both ITS models consistently demonstrated a detrimental long-term reversal of the mortality trend after the pandemic. While a precise level change was not apparent using traditional segmented regression, the ARIMAX-based analysis successfully isolated a significant acute and lagged effect (β = +0.211; p = 0.0029). We estimated 6,540 excess tuberculosis deaths in Brazil between 2020 and 2023 (95%CI 3,950 to 9,130). The forecasting model with the pandemic effect projected higher mortality rates from 2024 to 2030, while the counterfactual scenario showed a continued decline.

**CONCLUSIONS:**

The Covid-19 pandemic had a substantial negative impact on tuberculosis mortality in Brazil, representing a setback in achieving national and global elimination targets.

## INTRODUCTION

 Tuberculosis remains one of the most persistent and challenging infectious diseases globally, with a particularly heavy toll in low- and middle-income countries. Despite being preventable and curable, tuberculosis continues to affect millions of people each year, driven by social determinants such as poverty, undernutrition, overcrowded living conditions, and limited access to health care services^1^. 

 In its strategy, the World Health Organization (WHO)^1^ set a target of reducing global tuberculosis incidence by 90% and tuberculosis-related deaths by 95% by 2035, using 2015 as the baseline year. These goals are part of the End Tuberculosis Strategy, which emphasizes integrated patient-centered care, bold policies, and intensified research and innovation to eliminate tuberculosis as a public health threat. 

 According to the WHO^1^, Brazil is among the countries with a high burden of tuberculosis globally, characterized by high incidence, morbidity, mortality, and catastrophic costs for families. In alignment with the WHO’s End Tuberculosis Strategy, the Brazilian Ministry of Health^2^ set the goal of reducing the incidence to fewer than ten cases per 100 thousand inhabitants and tuberculosis mortality to fewer than one death per 100 thousand inhabitants within the same timeframe. 

 In recent decades, tuberculosis has remained the leading cause of death from a single infectious agent worldwide, although it was temporarily surpassed by Covid-19 between 2020 and 2022^1^. Despite this, the absolute number of tuberculosis deaths increased in 2020 and 2021, reaching 1.40 million and 1.42 million, respectively. In 2022, tuberculosis deaths declined to 1.32 million, and further decreased to 1.25 million in 2023 — below the pre-pandemic level of 1.34 million recorded in 2019^1^. 

 Evidence suggests that the Covid-19 pandemic disrupted health systems globally, leading to delays in tuberculosis diagnosis, treatment interruptions, and reduced access to essential services, which has reversed years of progress in tuberculosis control^3,4^. It is estimated that disruptions caused by the Covid-19 pandemic led to nearly 700 thousand excess tuberculosis deaths between 2020 and 2023, when compared to the number of deaths that would have been expected had pre-pandemic trends continued^1^. 

 In Brazil, a significant increase in the absolute number of tuberculosis deaths was observed in 2021 (5,120) and 2022 (5,845), compared to 2020 (4,569), suggesting the impact of the Covid-19 pandemic in tuberculosis mortality^5^. National reports confirm this descriptive rise; however, to our knowledge, no robust epidemiological study has employed advanced time-series analysis to statistically quantify and isolate the temporal impact of the pandemic event. The present study aimed to analyze changes in tuberculosis mortality trends in Brazil over recent decades, assess the temporal relationship between the Covid-19 pandemic and tuberculosis mortality using time series intervention models, estimate the associated excess tuberculosis deaths, and forecast future mortality trends. 

## METHODS

### Study Design and Population

 We utilized an ecological time series design to analyze tuberculosis mortality in Brazil from 2000 to 2023. Tuberculosis mortality data was extracted from the Brazilian Mortality Information System (SIM/Datasus), accessed in November of 2024, utilizing the International Classification of Diseases and Related Health Problems 10^th^ Revision (ICD-10) codes A15–19 as the reported cause of death. Only consolidated data were used for the entire study period. 

### Outcome Variables and Data Sources

 The outcome variable was the Age-Adjusted Tuberculosis Mortality Rate, calculated per 100 thousand people. Mortality rates were obtained by dividing the absolute number of tuberculosis-related deaths each year (SIM/Datasus) by the annual estimated population. These estimations were extracted from 2024 retrospective projections of the *Instituto Brasileiro de Geografia e Estatística* (Brazilian Institute of Geography and Statistics — IBGE), based on the 2022 Census^6^. Age-adjustment was performed using the WHO World Standard Population^7^. 

### Analysis Plan

#### Tuberculosis mortality trend analysis

 To identify significant shifts in the temporal trend of tuberculosis mortality, we applied joinpoint regression analysis. Joinpoint regression identifies points in time where significant changes occur in the trend of a time series, facilitating a better understanding of underlying patterns. It is graphically represented as straight line segments connected by junction points, which indicate significant changes in the trend of the time series. It employs a log-linear regression model to calculate the annual percentage change (APC) for each segment, with the slope representing the average yearly percentage change of that segment. The average APC (AAPC) describes the overall rate of change across the entire time series^8^. 

 By using tuberculosis-related mortality rates (dependent variable), along with their standard error and year (independent variable), we modeled the tuberculosis mortality time series in Brazil and its five macroregions, as well as by sex and age group. In the model, we used age-adjusted mortality, except for age group. For the latter, we used crude mortality rates, dividing by the following groups: 0–14, 15–59, and above 60 years. These age groups are the same as those used by the Brazilian Ministry of Health. A confidence interval of 95% (95%CI) was used for the APC and AAPC calculation. 

#### Covid-19 impact on tuberculosis mortality

 Interrupted time series (ITS) using segmented linear regression is a widely used method for evaluating public health interventions, particularly when assessing the impact of a single, well-defined event over a short period. However, when the effect of an intervention is lagged, progressive, or cumulative, alternative models may be more appropriate^9,10^. 

 While AutoRegressive Integrated Moving Average (ARIMA) models are commonly used for forecasting, they are also suitable for intervention analysis, especially when paired with binary variables representing structural breaks or disruptions. This approach aligns ARIMA with the intervention analysis framework proposed by Box and Tiao^11^, where intervention effects are modeled explicitly. This can be considered equivalent to an ITS based on ARIMA with eXogenous variables (ARIMAX), which incorporates exogenous variables — such as intervention dummies — into the modeling process. Compared to segmented regression, ARIMAX offers superior correction for serial autocorrelation and greater flexibility to accurately model complex temporal dependencies, including transient and cumulative impacts. This makes it a robust option for time series impact evaluation^10,12-14^. 

 To assess the impact of the Covid-19 pandemic on tuberculosis mortality, we defined the pandemic period as spanning from 2020 to 2022. Then, we explored two intervention timings: an immediate effect starting in 2020 and a one-year lagged effect starting in 2021. The decision to prioritize the one-year lag was driven by the descriptive data showing a clear increase in mortality starting in 2021, rather than 2020, and the clinical rationale that the chronic nature of tuberculosis causes progression to death to be typically delayed. 

 We first applied a segmented linear regression-based ITS (SLR-ITS) model, initially setting 2020 as the intervention year and subsequently testing 2021. The independent variables for this model were constructed as a continuous time variable, an intervention dummy (level change), and a post-intervention trend variable. To account for heteroscedasticity, all regression models were executed using weighted least squares. 

 Afterward, we employed an ARIMAX model to capture the potentially more complex temporal dynamics introduced by the pandemic. The ARIMAX model used the standardized mortality rate as the endogenous variable and a single exogenous variable (xreg) to represent the pandemic’s impact. In the one-year lag model, the variable was coded as 0 from 2000 to 2020 and as 1 for 2021, 2022, and 2023. The ARIMAX parameter estimation relied on maximum likelihood methods, which are robust to heteroscedasticity and explicitly model serial dependence, serving as the primary method for effect isolation. 

#### Excess tuberculosis deaths due to the Covid-19 pandemic

 Based on the ARIMA model fitted to pre-pandemic data, we estimated the number of tuberculosis deaths that would have occurred from 2020 to 2023 had the pandemic not happened. These projected mortality rates were converted into absolute numbers of deaths using population data and compared to the observed deaths during the same period. The difference between observed and projected deaths represents the excess mortality due to the Covid-19 pandemic, with 95%CIs. 

#### Tuberculosis mortality forecasting until 2030

 ARIMA modeling is well-regarded for epidemiological forecasting^15-17^. To project tuberculosis mortality trends in Brazil through 2030, we applied two time series models to generate comparative scenarios. First, the Counterfactual Scenario was established by fitting an ARIMA model to the pre-pandemic series (2000–2019), simulating the trajectory of mortality had the pandemic not occurred. Next, the Pandemic Impact Scenario utilized an ARIMAX model fitted to the full time series (2000–2023). This model incorporated a binary intervention variable (coded as 1 for the lagged effect period 2021–2023) representing the pandemic’s impact. For the forecast period (2024–2030), the exogenous variable was set to zero, based on the assumption that the acute external pulse concluded after 2023. Both models were validated using residual diagnostics, and the resulting forecasts included 95%CIs. 

### Statistical Analyses Tools and Ethical Aspects

 Statistical analyses were performed using the R software (Version 4.3.3) and the Joinpoint Regression Program (Version 5.2.0.0. April, 2024) from the US National Cancer Institute (NCI). This study relied exclusively on publicly accessible aggregated data and was thus not subject to review by a Research Ethics Committee. 

## RESULTS

**Figure 1:**
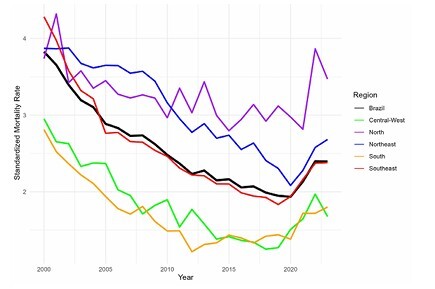
Age-adjusted tuberculosis mortality rate in Brazil and its regions, 2000–2023.

 A total of 117,022 tuberculosis-related deaths were reported from 2000 to 2023, with a male-to-female ratio of 2.9 to 1. Most deaths (60.5%) occurred among individuals aged 15 to 59 years (Table 1). Although the absolute number of deaths was higher in the Southeast region (45.1%), the overall mortality rate was highest in the North and Northeast regions, while it was lowest in the Central-West and South (Figure 1). 

**Table 1:** Tuberculosis death distribution in Brazil (2000–2023).

Variable		n	%
Sex			
	Male	87,023	74.4
	Female	29,963	25.6
Age group (years)			
	0–14	1,251	1.1
	15–59	70,071	60.5
	60 and older	44,582	38.5
Region			
	Northeast	36,359	31.1
	Central-West	5,580	4.8
	North	10,141	8.7
	South	12,193	10.4
	Southeast	52,749	45.1

 An increase in standardized mortality was observed starting in 2021 (2.13 deaths per 100 thousand inhabitants, compared to 1.93 deaths per 100 thousand inhabitants in 2020, respectively). This increase culminated in a mortality rate of 2.4 per 100 thousand inhabitants in 2022 and 2023, which is comparable to the rate observed in 2011 (2.36). 

### Tuberculosis Mortality Trend Analysis

**Figure 2:**
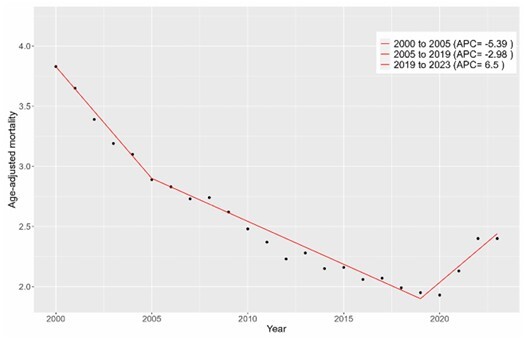
Trend of tuberculosis mortality rate in Brazil, 2000 to 2023 (Jointpoint). APC: annual percentage change.

 In the analysis of tuberculosis-related mortality in Brazil, we observed a two-joinpoint trend (three segments) as the best-fitting model. There was a significant decline in mortality from 2000 to 2019, with a steeper slope from 2000 to 2005 (APC = -5.39; p < 0.000001), followed by another significant decline from 2005 to 2019 (APC = -2.98; p = 0.0056). In contrast, there was a marked increase in mortality after 2019 (APC = 6.5; p < 0.000001). This reversal in trend, characterized as an inverted J-shaped curve, coincided with the Covid-19 pandemic. Considering the entire period, the AAPC was -1.93 (p < 0.000001) (Table 2 and Figure 2). 

**Table 2:** Best-fitted joinpoint model values of tuberculosis mortalitya time series in Brazil, 2000 to 2023.

Region			APC	95%CI	p-value
Brazil					
	2000–2005		-5.39	-8.54 to -3.99	< 0.001
	2005–2019		-2.98	-3.32 to -2.35	0.006
	2019–2023		6.50	4.42 to 9.98	< 0.001
	AAPC		-1.93	-2.19 to -1.69	< 0.001
Northeast					
	2000–2008		-1.07	-1.74 to -1.71	0.028
	2008–2012		-5.53	-7.65 to -3.78	< 0.022
	2012–2017		-1.67	-2.62 to 1.04	0.214
	2017–2020		-6.70	-8.49 to -4.46	< 0.001
	2020–2023		8.90	6.31 to 12.86	< 0.001
	AAPC		-1.51	-1.76 to -1.28	< 0.001
Central–West					
	2000–2018		-4.41	-5.26 to -3.72	< 0.001
	2018–2023		8.91	4.22 to 20.33	< 0.001
	AAPC		-1.66	-2.38 to -1.06	< 0.001
North					
	2000–2005		-7.87	-10.9 to -6.49	< 0.001
	2005–2019		-3.09	-3.46 to -2.71	< 0.001
	2019–2023		7.67	5.64 to 11.68	< 0.001
	AAPC		-2.38	-2.66 to -2.15	< 0.001
South					
	2000–2012		-5.80	-7.61 to -6.65	0.02
	2012–2020		1.29	-6.56 to 2.92	0.494
	2020–2023		8.48	2.66 to 15.89	< 0.001
	AAPC		-1.60	-2.18 to -1.18	< 0.001
Southeast					
	2000–2005		-7.87	-10.90 to -6.49	< 0.001
	2005–2019		-3.09	-3.46 to -2.71	< 0.001
	2019–2023		7.67	5.64 to 11.68	< 0.001
	AAPC		-2.38	-2.66 to -2.15	< 0.001
Sex					
	Male				
		2000–2005	-5.14	-8.25 to -3.80	< 0.001
		2005–2019	-2.79	-3.13 to -2.15	0.005
		2019–2023	6.18	4.15 to 9.67	< 0.001
		AAPC	-1.81	-2.06 to -1.58	< 0.001
	Female				
		2000–2011	-4.61	-8.05 to -3.64	0.037
		2011–2020	-2.73	-4.20 to 0.77	0.079
		2020–2023	10.18	4.47 to 18.42	< 0.001
		AAPC	-2.05	-2.58 to -1.64	< 0.001
Age group					
	0–14 years				
		2000–2002	-16.68	-21.63 to -6.60	< 0.001
		2002–2016	-4.92	-7.06 to 1.18	0.069
		2016–2023	9.32	4.24 to 26.09	0.016
	15–59 years				
		2000–2005	-5.48	-8.75 to -4.00	< 0.001
		2005–2019	-1.77	-2.24 to -1.17	0.002
		2019–2023	8.58	6.27 to 12.48	< 0.001
		AAPC	-0.87	-1.15 to -0.63	< 0.001
	≥ 60 years				
		2000–2020	-3.45	-3.69 to -3.24	< 0.001
		2020–2023	6.76	3.38 to 12.80	< 0.001
		AAPC	-2.18	-2.42 to -1.99	< 0.001

APC: annual percentage change; AAAPC: average annual percentage change; 95%CI: 95% confidence interval; ^a^Age-adjusted mortality for region and sex; crude mortality for age group.

 Although tuberculosis mortality among men was significantly higher, there was no significant difference in the trend curves between the sexes. In the age-stratified analysis, we observed a similar abrupt change in the mortality trend: from a significant negative to a significant positive slope. However, for the 0–14 years group, this reversal pattern occurred earlier, starting after 2016, while in the 15–59 years and over 60 years groups, it occurred after 2019 and 2020, respectively (Table 2). 

 In the analysis of each Brazilian macroregion, only the Northeast exhibited a four-joinpoint best-fitted model, while the others had one or two joinpoints. Four macroregions showed a declining trend of tuberculosis mortality during most of 2010s, except for the South, where the decline halted as early as 2012. All macroregions exhibited a steep positive slope by the end of the analyzed period (Table 2). 

### Covid-19 Impact on Tuberculosis Mortality

 Both SLR-ITS models tested (interruption points 2020 and 2021) confirmed a statistically significant decreasing trend in standardized tuberculosis mortality rates during the pre-pandemic period (β = -0.08; p < 0.001). No immediate level change was detected following the pandemic’s onset in either 2020 (β = -0.023, p = 0.862) or 2021 (β = 0.259, p = 0.174). However, both models revealed a significant change in the post-intervention slope, indicating a reversal of the mortality trend, with slopes of β = 0.254 (p < 0.001) from 2020 onwards and β = 0.217 (p = 0.019) from 2021 onwards. Both models exhibited a robust fit (Adjusted R2 > 0.91) and satisfied the assumption of non-autocorrelated residuals (Breusch-Godfrey test: p > 0.05). 

 We then assessed the intervention timing using two ARIMAX specifications. We first tested an immediate effect starting in 2020, but the intervention variable was not statistically significant (β = -0.103; p = 0.210). This initial model (ARIMA [1,2,0]) was nonetheless methodologically sound, exhibiting a favorable Akaike Information Criterion — AIC (-31.92) and robust residuals (Ljung-Box test: p = 0.398). Subsequently, assuming a one-year lagged effect, the final ARIMAX model was selected, specified as ARIMA (1,2,0) with one exogenous variable representing the pandemic effect. The model demonstrated a statistically significant impact (β = +0.211; p = 0.003), suggesting a measurable deviation from the baseline mortality trend in the subsequent years. The model exhibited a strong fit, with a favorable AIC (-37.09), low residual error (Root Mean Squared Error — RMSE = 0.085), and uncorrelated residuals (Ljung-Box test: p = 0.565). 

### Excess Tuberculosis Deaths due to the Covid-19 Pandemic

 From 2020 to 2023, a considerable excess of tuberculosis deaths was observed when comparing recorded data with the counterfactual forecast derived from the pre-pandemic model. The contrafactual scenario was based on a validated ARIMA (1,2,0) model fitted to the 2000–2019 series, with residuals confirmed to be white noise (Ljung-Box test: p = 0.403). The calculated excess was 594 deaths in 2020 (95%CI 292 to 896), and 1,220 in 2021 (95%CI 731 to 1,709). The burden accelerated in the subsequent years, reaching 2,264 deaths (95%CI 1,506 to 3,022) in 2022 and 2,462 deaths (95%CI 1,421 to 3,503) in 2023. The cumulative total excess death for the period was 6,540 (95%CI 3,950 to 9,130). 

### Tuberculosis Mortality Forecasting until 2030

**Figure 3:**
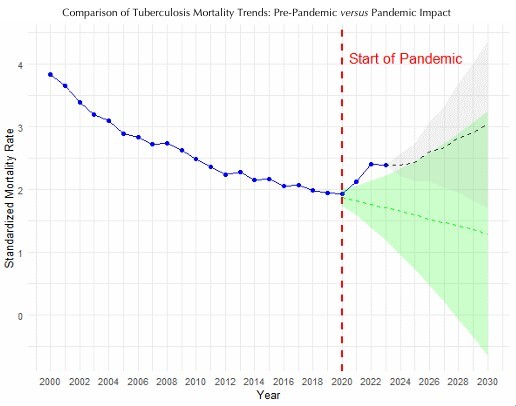
Comparison of tuberculosis mortality forecasts: counterfactual (pre-pandemic trend) *versus* actual pandemic impact.

 The comparison of forecasts with and without the pandemic effect revealed divergent scenarios. The model incorporating the pandemic effect projected higher mortality rates from 2024 to 2030, while the counterfactual scenario, assuming no pandemic, showed a continued decline in mortality between 2020 and 2030. Despite the exclusion of the exogenous variable, the forecast shows a sustained increase in mortality up to 2030. This phenomenon is a direct consequence of the model’s structure (ARIMA [1,2,0]), where the second-order differentiation (d = 2) internalizes the dramatic positive acceleration observed during the pandemic (Figure 3). 

## DISCUSSION

 Between 2000 and 2023, tuberculosis mortality in Brazil decreased at an average annual rate of 1.93%. This declining trend, however, was offset by a reversal from 2021 to 2023, when mortality rose by 6.5% per year, coinciding with the Covid-19 pandemic. No statistically significant effect of the pandemic in reverting the decline of tuberculosis mortality was detected using traditional ITS analysis. However, the ARIMAX-based ITS identified a significant pandemic-related lagged effect (β = 0.211; p = 0.0029), leading to an estimated 3 thousand excess tuberculosis deaths between 2020 and 2023. The forecasting model incorporating the pandemic effect projected higher mortality rates from 2024 to 2030, while the counterfactual scenario predicted a continued decline in mortality between 2020 and 2030, had the pandemic not occurred. 

 A declining trend in tuberculosis deaths in Brazil has been observed since at least the 1980s. Bierrenbach et al.^18^ reported a decrease from 5.8 deaths per 100 thousand inhabitants in 1980 to 2.8 in 2004. Despite the significant reduction in mortality during this period, the decline was not linear and experienced some setbacks (i.e., an apparent stationary trend from 1985 until the end of the 1990s), likely associated with the progression of the acquired immunodeficiency syndrome (AIDS) epidemic in major urban centers. This overall decline can be attributed to improvements in social indicators, including cash transfer programs, as well as advances in primary health care^19,20^. 

 After a prolonged period of decline in tuberculosis mortality, a noteworthy reversal was observed starting in 2021, amid the Covid-19 pandemic. Our joinpoint model identified this as a shift in the downward trend in 2019, reversing to a significant upward trend through 2023 across all analyzed categories (macroregion, sex, and age groups). However, in certain subgroups, the declining trend was interrupted earlier in the 2010s; for example, the Southern Region showed a stationary trend, and the 0–14 years group exhibited an increasing trend. The precise reasons for these premature reversals remain unclear. Hypotheses suggest that the stabilization in the Southern Region may reflect a "saturation effect" due to historically favorable socioeconomic indicators, while the early rise in vulnerable groups, such as children, may be linked to heightened sensitivity to the 2010s economic crisis. 

 By applying SLR-ITS analysis, we did not detect a statistically significant immediate change in tuberculosis mortality trends following the onset of the Covid-19 pandemic. However, the significant negative and positive slopes observed before and after the intervention point, respectively, may still suggest an indirect pandemic-related effect. The inability of SLR-ITS to detect a precise change point may underscore its limitations in capturing delayed or cumulative impacts of external shocks. In contrast, the ARIMAX model identified a statistically significant coefficient associated with the pandemic period, providing stronger evidence of its influence on tuberculosis mortality. 

 It is reasonable to assume a relationship between tuberculosis mortality during the analyzed period and the frequency of new cases. In fact, since the economic recession that began in 2015, there has been an increasing trend in tuberculosis incidence. Silva and Galvão^21^, using SLR-ITS, found a significant decline in monthly tuberculosis incidence from 2001 to 2014 (β = -0.005; p < 0.001), followed by an increase from January 2015 to March 2021 (β = 0.013; p < 0.001). Li et al.^22^ had previously noted this trend reversal starting in 2015, estimating an excess of 22,900 tuberculosis cases from 2015 to 2019, using the period from 2010 to 2014 as a baseline. However, no statistically significant excess in tuberculosis mortality was found for the same period. 

 Like most countries worldwide, Brazil experienced a sharp decline in tuberculosis notifications in 2020 (from 37.3 cases per 100 thousand inhabitants in 2019 to 32.7 in 2020) due to the Covid-19 pandemic. This decline was followed by a rebound in the incidence rate in 2022 and 2023 (38 and 37 cases per 100 thousand inhabitants, respectively)^5^. It is speculated that this rebound, also observed on a global scale^1^, is due to late notifications and a real increase in tuberculosis cases. In other words, it may reflect not only delayed diagnosis but also an increase in community transmission during this period^1^. 

 As suggested, the increase in tuberculosis mortality is likely attributable to a combination of two concurrent factors: delays in diagnosis and treatment, which led to more advanced stages of the disease, and the concurrent rise in tuberculosis incidence during the pandemic. The negative impact of the Covid-19 pandemic on tuberculosis indicators exemplifies the concept of syndemia, which refers to the synergistic interaction of multiple pathological conditions exacerbated by social, economic, and environmental factors^23^. Socioeconomic factors may have exacerbated the worsening indicators of a disease closely linked to social determinants. According to IBGE, poverty and extreme poverty rates reached 36.7 and 9% of the Brazilian population in 2021, respectively, based on World Bank criteria. For comparison, these rates were 30.8 and 5.2% in 2014^24^. 

 The cumulative excess tuberculosis deaths attributable to the pandemic event (6,540; 95%CI 3,950 to 9,130) reached a magnitude higher than the average annual mortality of the prepandemic period from 2010 to 2019 (4,550 deaths per year). This means that, taking into consideration the confidence interval, the pandemic effectively imposed the burden of around one up to two ‘extra years’ of tuberculosis deaths. 

 In our study, forecasting tuberculosis mortality trends up to 2030, with and without accounting for the pandemic period, yielded divergent trajectories. The model including the pandemic effect projected an upward trend — reflecting the cumulative impact of the pandemic rather than the ongoing influence of the exogenous variable, which was deactivated after 2023. In contrast, the counterfactual scenario indicated a continued decline. However, both models produced confidence intervals that encompassed increasing and decreasing trends, highlighting the uncertainty inherent in long-term projections based solely on time series data. 

 This study has several limitations. Firstly, it was based on secondary data. Although microbiological confirmation of tuberculosis is commonly available in cases reported to the Notifiable Diseases Information System, such confirmation is often absent in the SIM. This discrepancy stems from the lack of automated linkage between the two databases, raising concerns about potential misclassification in mortality records. However, this limitation is likely consistent throughout the entire time series and, therefore, is unlikely to substantially bias trend estimates. 

 Another limitation in our ITS analysis is our short series (n = 24 annual observations). This issue was mitigated by selecting a highly parsimonious model specification (ARIMA [1,2,0]) guided by the AIC. This structure was statistically adequate, as confirmed by the Ljung-Box test, indicating that the model successfully captured the underlying temporal dynamics and avoided overfitting. Besides that, an inherent limitation in ITS design is the difficulty in establishing causal attribution in the face of unmeasured concurrent events. To refine future predictions and enable health planning, modeling efforts could include relevant socioeconomic, programmatic, and policy variables (i.e., other exogenous regressors). 

 Despite limitations, the ARIMAX model proved to be a valuable methodological approach for capturing more complex and potentially lagged effects of external interventions on longitudinal outcomes. This model allowed us to isolate the delayed impact of the Covid-19 pandemic on tuberculosis mortality, accounting for the temporal structure of the data more effectively than traditional SLR-ITS. 

 Finally, the reversal of the tuberculosis mortality trend poses a setback to the goal of eliminating the disease by 2035, mandating intensified efforts for disease control. While the Covid-19 pandemic may have been an exceptional public health event that only temporarily affected the historical mortality trend for tuberculosis, it also highlights existing vulnerabilities in policy, programmatic strategies, health system capacity, and socioeconomic factors that could hinder the return to a sustained reduction in tuberculosis cases and deaths in Brazil. 

## Data Availability

The data are available upon request to the corresponding author.
